# The Alien in America

**Published:** 1905-12-30

**Authors:** 


### THE ALIEN IN AMERICA.

The tatements made about the alien workman—his long hours of work and his low standard of living—are, in this country, often made vaguely, without much knowledge of the facts of the case, for here we have no governmental bureau whose work it is to investigate these points. But the bureau of labour of the United States has thought it worth while to inquire into the ways of living of its workers, both native and foreign, and from the comparison between these some facts may be observed which have a value even on this side of the Atlantic. America is the bright occidental star which draws towards it those in every European country who are discontented with their lot. Thus its alien question is a much larger one than any we have yet had to cope with, and from its experience we may be able to infer, not indeed the extent to which the alien displaces native labour, but the way in which he spends what he earns. The reports in question were obtained from over 25,000 families of all nationalities living in the principal industrial localities of 33 States. They may therefore be taken as fairly representative.

The first thing to be noted is the amount earned by each of these typical families. And here one finds a fact that may cheer certain pessimists among us. Clever and energetic as is the native-born inhabitant of "the States," some foreigners earn more than he. And first among these foreigners are (in order of earnings) Scotch, English, Welsh, and Irish. After these come Canadians, Norwegians, and Swiss, but the Dutch are not far behind, and have the distinction of spending a smaller proportion of their earnings than any other nationality. The most extravagant in this respect are those who earn least—the Austro-Hungarians and Russians. But this need not be set down to their discredit, for, the primary needs of existence being the same for all, it follows that in the matter of necessaries the largest proportionate—though not absolute—expenditure must come from the smallest income. But it must be owned, on the other hand, that the foreigners depend more than the native on the aid given to the family income by children. Thus we find that of the German families whose incomes are dealt with, more than 33 per cent. of the families have children at work, and of the Irish over 30, while the Welsh top the list with 35 per cent. Of all children those of Swedish parents seem to be the most fortunate. Only 10 per cent. of the Swedish families get any help from their children, while the native American is helped by his children's work in the proportion of 18 per cent. The latter proportion, or a fraction less, holds good of the French families, but in this nation Madame has always been in a commercial sense the help-mate of Monsieur, and so she remains although removed to another hemisphere. Among wives who work, France easily heads the list with 13 per cent., while among the native born the percentage is nearly 9. Among German, Irish, and Italian wives the percentage is a very little higher. Of English and Scotch wives only about 6 per cent. work for wages, while the wives of Welshmen work only in the proportion of 1·71 per cent. But of all the home-staying folks the Dutchwoman takes the lead. Not one per cent. of the women from the Netherlands works for wages, though these same ladies run the French woman close in taking lodgers or boarders. The proportion of families taking in strangers is, of French 29, and of Dutch 28. One would be inclined to set this down as a testimony to cookery and cleanliness if one did not find that lodgers are more common in Irish families than in any others, and neither of these domestic virtues are reckoned as being common among the daughters of the Emerald Isle. In this connection it is curious to note that a much larger proportion of the family income is derived from this source in Irish families than in any others. Doubtless the explanation is the instinct that makes people of every nationality make their home with those of their own country, an instinct which is, perhaps, stronger in the Celtic races than in any other.

We have said that comparatively few Dutchwomen go out to work, but when they do the results are such as to justify the step. Alone among working wives, the woman of the Netherlands earns more than her husband—only about 5s. more in the year, it is true, but that is quite enough to give her a satisfactory feeling of superiority. Can one connect with the economic energy of the woman of the Netherlands the fact that of the Dutch families more than 46 per cent. own their own homes? The Dutchwoman certainly sees in that the result of her labours. Next to this nation in home love, at least as exemplified in the desire and ability to possess a house of their own, come the Scandinavians—first Norwegians, of whom 38 per cent. own their homes, next Danes, and then Swedes. The kind of home should, however, be taken into consideration in deciding on the merit of seeking to possess one of one's own. It is better for the health of a family to rent a house of sufficient size than to save to buy one that lacks proper accommodation. Here it must be owned that the Englishman scores. The allowance of rooms per family is rather smaller than that of the Dutchman, but then the family is smaller too, and the allowance of rooms per individual—1·13—indicates a comfortable amount of elbow-room. Compare with that the Italian who allows only ·77 of a room for one person, or the Russian and Austrian whose idea of the requisite space is only a little higher. It might be expected as a consequence that the families of English birth would expend a larger proportion of their income in rent than those of other nationalities. But the Englishman is only second in this expenditure: Ireland tops the list, which would seem strange, seeing that the Irishman is content with something less than a room for each member of the family, if one did not remember that superabundance of lodgers which helps out his income. In expenditure on clothing England also comes second, but here it is the French who are—should one say naturally?—the most extravagant, while of all the nationalities, those of French extraction spend the smallest proportion on food. The most extravagant in that respect would appear to be the Austrians and Russians, but these two and the Italian are the nations whose emigrants earn least in the country whither they have gone, and doubtless they do not really spend so much as members of the more capable and fortunate nations.

If one may draw from the experience of America any estimate as to the kind of immigrants whom, having regard to the welfare of our country, we ought to encourage here, one would give the preference to the Scandinavians, Dutch, and French. Germans, with all their undeniable good qualities, encourage child labour too much, while Italians, Austrians, and Russians, seem by their comparatively small earning power to belong to the ranks of unskilled labour, which it is never desirable to swell, and incline to huddle together in conditions which must inevitably be overcrowded and therefore insanitary.

